# Delayed FDG PET Provides Superior Glioblastoma Conspicuity Compared to Conventional Image Timing

**DOI:** 10.3389/fneur.2021.740280

**Published:** 2021-11-16

**Authors:** Jason Michael Johnson, Melissa M. Chen, Eric M. Rohren, Sujit Prabhu, Beth Chasen, Osama Mawlawi, Ho-Ling Liu, Maria Kristine Gule-Monroe

**Affiliations:** ^1^Department of Neuroradiology, University of Texas MD Anderson Cancer Center, Houston, TX, United States; ^2^Department of Radiology, Baylor College of Medicine, Houston, TX, United States; ^3^Department of Neurosurgery, University of Texas MD Anderson Cancer Center, Houston, TX, United States; ^4^Department of Nuclear Medicine, University of Texas MD Anderson Cancer Center, Houston, TX, United States; ^5^Department of Imaging Physics, University of Texas MD Anderson Cancer Center, Houston, TX, United States

**Keywords:** PET CT scan, delayed imaging, glioblastoma, FDG (18F-fluorodeoxyglucose)-PET/CT, brain tumor

## Abstract

**Background:** Glioblastomas are malignant, often incurable brain tumors. Reliable discrimination between recurrent disease and treatment changes is a significant challenge. Prior work has suggested glioblastoma FDG PET conspicuity is improved at delayed time points vs. conventional imaging times. This study aimed to determine the ideal FDG imaging time point in a population of untreated glioblastomas in preparation for future trials involving the non-invasive assessment of true progression vs. pseudoprogression in glioblastoma.

**Methods:** Sixteen pre-treatment adults with suspected glioblastoma received FDG PET at 1, 5, and 8 h post-FDG injection within the 3 days prior to surgery. Maximum standard uptake values were measured at each timepoint for the central enhancing component of the lesion and the contralateral normal-appearing brain.

**Results:** Sixteen patients (nine male) had pathology confirmed IDH-wildtype, glioblastoma. Our results revealed statistically significant improvements in the maximum standardized uptake values and subjective conspicuity of glioblastomas at later time points compared to the conventional (1 h time point). The tumor to background ratio at 1, 5, and 8 h was 1.4 ± 0.4, 1.8 ± 0.5, and 2.1 ± 0.6, respectively. This was statistically significant for the 5 h time point over the 1 h time point (*p* > 0.001), the 8 h time point over the 1 h time point (*p* = 0.026), and the 8 h time point over the 5 h time point (*p* = 0.036).

**Conclusions:** Our findings demonstrate that delayed imaging time point provides superior conspicuity of glioblastoma compared to conventional imaging. Further research based on these results may translate into improvements in the determination of true progression from pseudoprogression.

## Introduction

Glioblastomas are infiltrative, malignant, and often terminal tumors of the central nervous system (1). Glioblastoma has a reported 1-year survival rate of 41.4% and a median survival of 6 months after the first recurrence (2). MRI is the dominant imaging type for central nervous system lesion and is utilized for initial diagnosis, biopsy guidance, surgery and radiation planning, response assessment, regular follow-up, and in the suspicion of recurrence (3). The standard treatment for glioblastoma is greatest safe surgical resection followed by concurrent chemotherapy and radiation therapy, followed with adjuvant temozolomide (4). Disruptions in the blood-brain barrier following treatment is associated with regional enhancement with gadolinium contrast-enhanced MRI. This phenomenon makes confident discrimination concerning true progression and treatment-related changes (pseudoprogression) based on MRI alone difficult and often impossible, even with advanced MRI techniques including functional and perfusion MRI (5). To further complicate the diagnostic challenge, treatment-related changes and residual or recurrent disease often coexist as seen in “mixed” pathology specimens (6).

Glioblastomas are known to exhibit significant hypermetabolic activity on FDG PET imaging (7). Unfortunately, the uptake and utilization of glucose by background normal brain parenchyma is also high such that it can be challenging to differentiate tumors from normal tissue (8). This issue does not consistently propose a significant pre-treatment diagnostic challenge. However, it does limit the diagnostic accuracy of FDG PET in the post-treatment brain when assessing for progressive disease vs. treatment-related changes. The lack of a significant lesion to background (L/B) ratio in FDG PET at the conventional 1-h imaging time point has resulted in the limited use of this imaging modality despite its potential for accurately identifying tumor vs. treatment-related change in the post-treatment setting.

A simple technique that has been successfully applied in small published trials (9–13) and at our institution is delayed FDG PET imaging. This type of imaging protocol can accentuate differences in the metabolic activity of the neoplastic and non-neoplastic brain. Delayed time point intervals have been published for small numbers of patients between ~ 1 and 8 h after the administration of FDG. Separation of disease from brain parenchyma appears to be highest at more prolonged delays. However, with a 110-min radiopharmaceutical half-life, longer delays lead to a progressive decrease in the degree of FDG activity and possibly issues with patient compliance. The prior small trials were generally successful in improving non-invasive diagnostic accuracy in a range of glioma grades and metastatic disease, but the optimal acquisition timing was not defined. The number of patients with glioblastoma in these studies was also small and included both treated and untreated lesions, which leads to a problem with generalizing prior work toward an idealized protocol for prospective usage.

Various trials have shown the diagnostic superiority of amino acid radiopharmaceuticals (for example, 11C-Methionine, O-(2-[18F]fluoroethyl)-L-tyrosine (18F-FET), and 3,4-dihydroxy-6-(18)F-fluoro-l-phenylalanine ((18)F-FDOPA)) to 18F-FDG; however, these tools are expensive (per dose rates can extend upwards of $3,000/dose), not readily available in most US centers, and none are currently approved by the United States Food and Drug Administration for glioma imaging (14). FDG is FDA approved for glioma imaging, readily available in the United States, and cost-efficient with a dose cost typically on the order of USD 500 (15). Given these practical and economic limitations to the widespread utilization of amino acid radiopharmaceuticals, we sought to optimize the diagnostic parameters that maximize the conspicuity of untreated glioblastoma with 18F-FDG. Identifying whether delayed time point imaging is superior to conventional imaging time points would provide useful information in the design of future trials assessing the value of 18-FDG for post-treatment progression vs. treatment-related changes (pseudoprogression/radiation necrosis) as well as more meaningful trials comparing 18-FDG to amino acid radiopharmaceuticals.

## Methods

### Patient Selection

Data were acquired in a prospective clinical imaging trial (clinicaltrials.gov, NCT02885272). The study is approved by The University of Texas MD Anderson Cancer Center institutional review board, compliant with all Health Insurance Portability and Accountability Act regulations, and maintains participant informed consent. Sixteen adult patients with suspected or biopsy-proven glioblastoma were enrolled in the study and received study imaging. Patients without pathologic confirmation of glioblastoma were enrolled based upon study neuroradiologist and neurosurgical consensus review of MRI that the most likely diagnosis for a new, solitary, intraaxial, enhancing lesion of at least 10 mm in size, in a patient without a history of cancer, was glioblastoma.

Patients with surgical resection of more than 25% of their glioblastoma at presentation to our center were excluded from the study. To avoid confounding factors in cerebral FDG PET imaging, patients with a history of prior brain malignancy, prior whole brain radiation, significant cerebrovascular disease, dementia, or prior traumatic brain injury were excluded. Patients with a known allergy to FDG or gadolinium-based contrast agents or a fasting blood glucose > 200 mg/dl as well as pregnant patients and children were also excluded.

### Imaging Protocol

Patients were scanned with a separate FDG PET CT and a 3.0 Tesla MRI (GE Discovery MR750 3T) examinations before maximal safe tumor resection. The PET and MRI examinations were performed on the same day. Patients were instructed to fast for at least 6 h before their imaging appointment. After the placement of an intravenous catheter and a blood glucose level check, patients were injected with 10.2 ± 1.2 mCi (standard of care) of FDG administration. No incident of significant hyperglycemia (glucose >200 mg/dl) was identified at the time of imaging. Three FDG PET CT imaging time points were performed on a GE Discovery 690 FX PET/CT (GE Healthcare, Waukesha, WI), at 64 min ± 6 min (“1-h”), 309 ± 24 min (“5-h”) and 475 ± 19 min (“8-h”) post FDG administration.

For patients without pathology proven glioblastoma, a body (eye through thigh) PET/CT was also performed at the 1-h time point to exclude other sites of malignancy. For the 1-h time point, PET/CT images of the brain were acquired for 10 min with LIST mode. The 5- and 8-h time points were acquired for 20 and 30 min, respectively. Patients were instructed to minimize activity between PET scanning time points. The CT component of the scan for each time point was acquired with a low dose technique at 120 kVp, 100 mA, pitch 0.98, and CTDI vol 4.02 mGy.

To standardize food intake and its potential impact on cerebral FDG distribution on the delayed time point images, patients were asked to adhere to a low carbohydrate, high protein meal following their 1- and 5-h time point PET scans. Patients were provided with information to assist them in food choices during their breaks. Significant findings, as determined by the study's PET readers, were communicated to the patient's referring neurosurgeon for potential further evaluation. One patient with glioblastoma was found to harbor a second primary malignancy (human papillomavirus virus-positive oropharyngeal squamous cell carcinoma). No other significant extracranial finding was identified on body PET examinations.

### Image Processing

The acquired PET data were reconstructed using iterative techniques with resolution recovery. All delayed time points were registered using rigid techniques to the 3D T1-post contrast imaging performed on the same day. Regions of interest (ROI) around the enhancing lesion were drawn, and the maximum standardized uptake value (max SUV) for the ROI was recorded. A region was also drawn on a mirrored site in the contralateral brain for calculation of the tumor to background ratio. This ROI incorporated both cortex and white matter. These values were then plotted vs. time and the time point corresponding to the highest value for each metric recorded.

### Subjective Image Analysis

Two experienced diagnostic radiologists in PET neuroimaging (one fellowship-trained in Nuclear Medicine, one with a Certificate of added qualification in neuroradiology) performed a subjective analysis of each imaging time point based on previously described methodology (16). Each PET imaging time point was viewed with fusion to the concurrent low-dose CT using a nuclear medicine imaging program (MIM Software, Beachwood, OH, USA). The two radiologists were instructed to manually optimize the window width and window level of the image to maximize the tumor conspicuity. Readers were blinded to all clinical information, including surgery/biopsy and outcome data. The FDG uptake was graded visually using the scheme demonstrated in Table 1. Magnetic resonance imaging data were not provided for review to avoid potential bias.

**Table 1:** Schifter FDG PET primary brain tumor visual analysis scale.

**Score**	**Finding**
1	Tumor uptake is less than contralateral white matter.
2	Tumor uptake equal to contralateral white matter.
3	Tumor uptake between contralateral white and gray matter.
4	Tumor uptake equal to contralateral gray matter.
5	Tumor FDG uptake is greater than the contralateral gray matter.

*Adapted from reference (13)*.

### Statistical Analysis

Statistical analysis for the objective analyses was performed using Stata version 11.2 software (Stata Corp., College Station, TX, USA) with a Wilcoxon signed-rank test. The interrater reliability for the subjective rater analysis was performed using Cohen's Kappa statistic. Statistical significance for this study was set at *p* < 0.05.

## Results

Sixteen patients with glioblastoma were enrolled, including nine men and seven women with a mean age at the time of study imaging of 64 ± 10 years (range 39–79). All 16 patients were isocitrate dehydrogenase (IDH) wild type, as tested by immunohistochemistry. Seven of the glioblastoma patients were negative for O6-methylguanine-DNA methyltransferase (MGMT) promoter methylation, four were positive for methylation, and five were not tested. MGMT testing was performed by pyrosequencing. Four patients were enrolled based upon biopsy results revealing glioblastoma, and 12 patients were enrolled based on their pre-operative imaging appearance.

**Figure 1:**
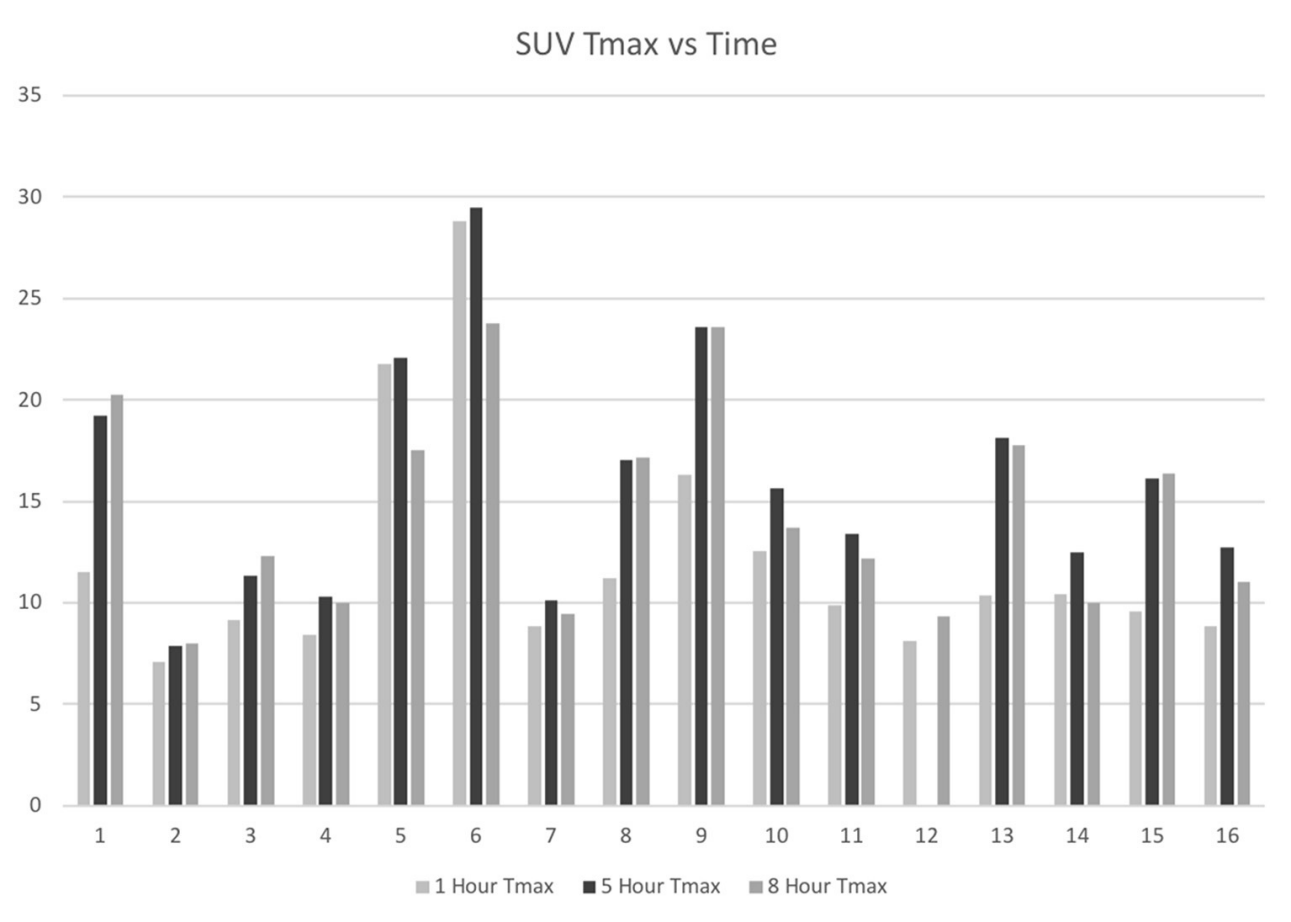
IDH-mutant, glioblastoma F18-FDG tumor maximum SUV at 1-, 5-, and 8-h following F18-FDG injection.

Fifteen of the 16 patients completed all three PET imaging time points, and all patients completed at least two-time points. One patient missed the 5 h imaging time point due to an unexpected conflict with another appointment but accomplished the 1 and 8 h time points. The mean maximum SUV (Tmax) for the tumors at 1, 5, and 8 h was 12.1 ± 5.7, 16.0 ± 5.8, and 14.5 ± 5.1, respectively (Figure 1). The Tmax for the lesions was statistically significantly increased for the 5 h time point over the 1 h time point (*p* = 0.0001), the 8 h time point over the 1 h time point (*p* = 0.0002), and the 8 h time point over the 5 h time point (*p* = 0.0015).

**Figure 2:**
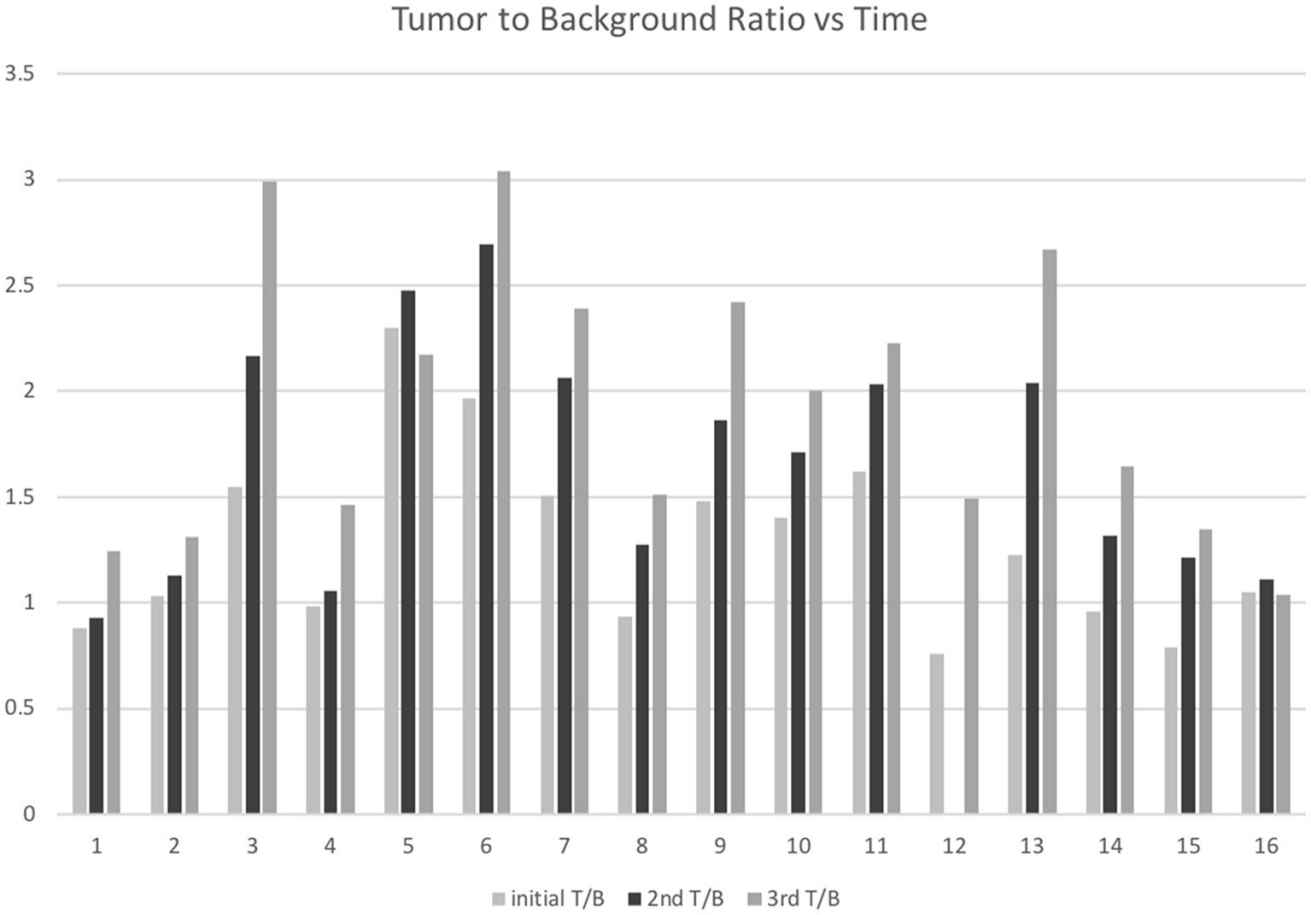
F18-FDG tumor to background ratios 1-, 5-, and 8-h following F18-FDG injection in 16 patients with IDH-wild type, glioblastoma.

The mean contralateral normal appearing brain maximum SUV (background) at 1, 5, and 8h was 9.6 ± 2.7, 10.2 ± 3.9, and 8.0 ± 3.3, respectively. The tumor-to-background ratio (T/B) for the tumors at 1, 5, and 8 h was 1.4 ± 0.4, 1.8 ± 0.5, and 2.1 ± 0.6, respectively. At the 1 h time point six tumors had T/B ratios <1. The number of tumors with T/B ratio <1 dropped to one at the 5 h time point and was zero at the 8 h time point (Figure 2).

There were statistically significant increases in tumor to background (T/B) ratios, also seen as 5 h over 1 h (*p* = 0.0001), 8 h over 1 h (*p* > 0.0002), and 8 h over 5 h (*p* = 0.0015).

**Figure 3:**
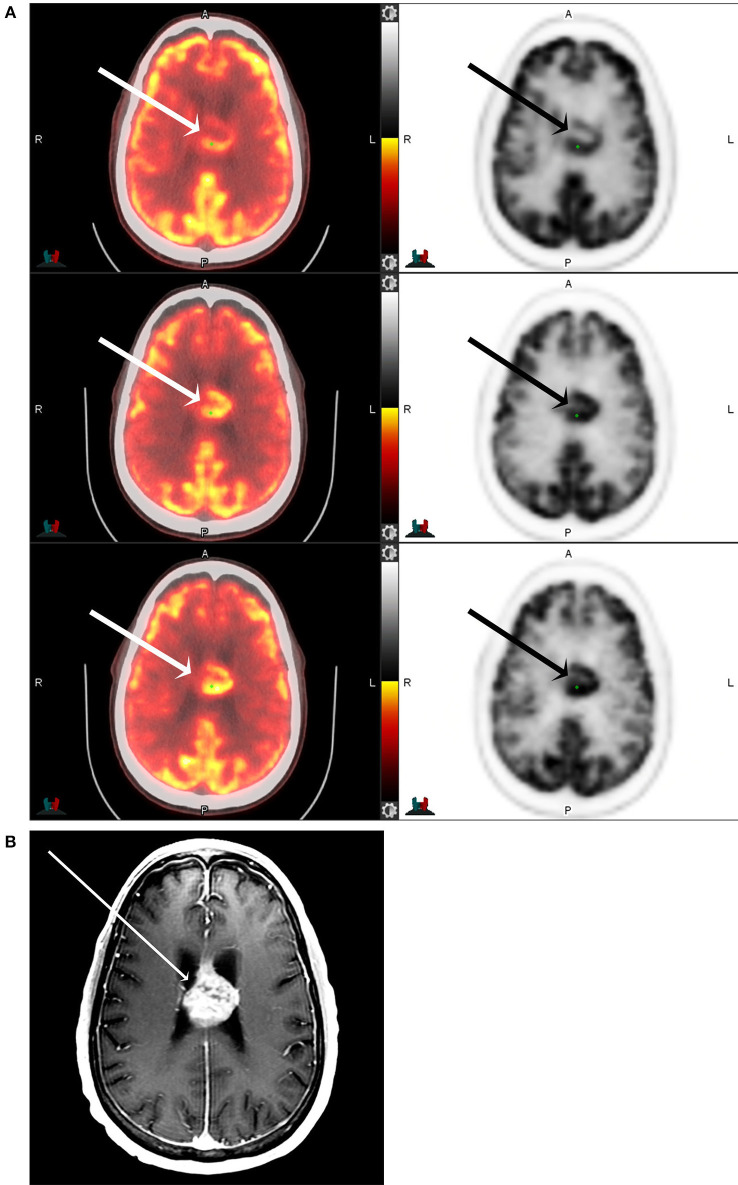
F18 FDG PET CT **(A)** and T1 postcontrast MRI of the brain **(B)** of a 71-year-old male with IDH-mutant, glioblastoma involving the corpus callosum and cingulate gyrus (arrows) at 1-, 5-, and 8-h following F18-FDG injection. The maximum tumor SUV, as well as the tumor to background ratios, are seen to increase with increasing time from the injection.

**Figure 4:**
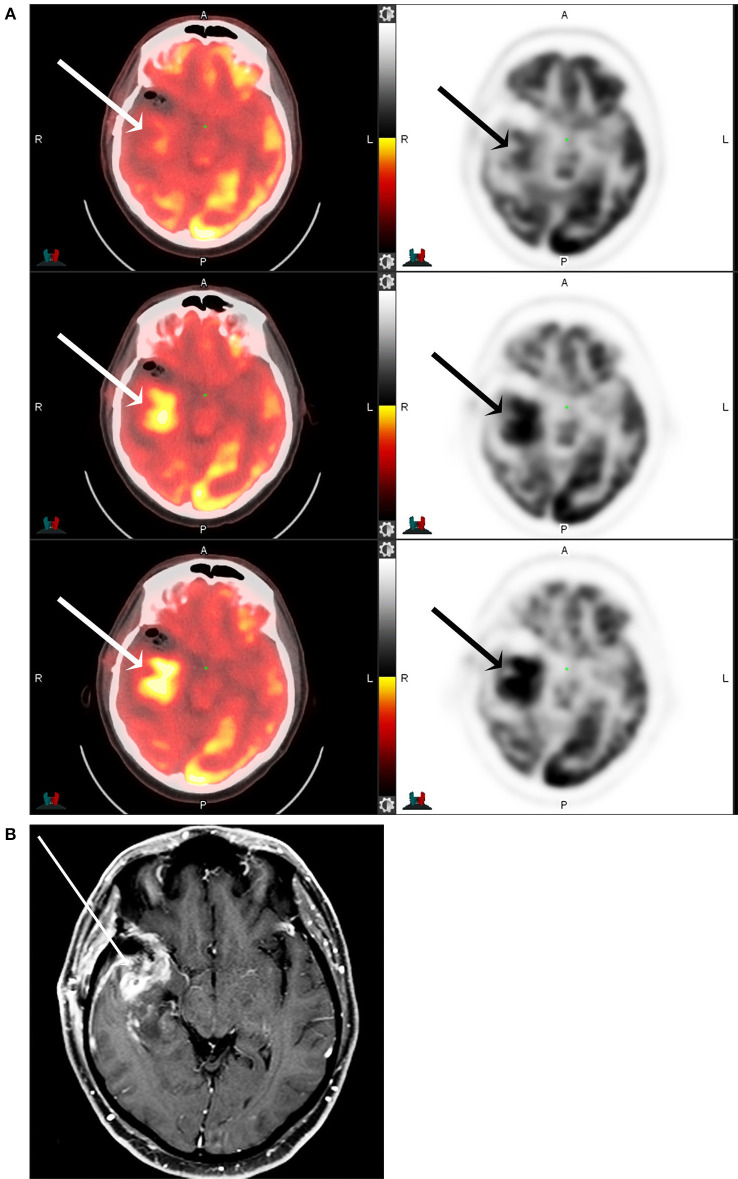
F18 FDG PET CT **(A)** and T1 post-contrast MRI of the brain **(B)** of a 53-year-old male with partially resected IDH-mutant, glioblastoma of the right anterior temporal lobe (arrows) at 1-, 5-, and 8-h following F18-FDG injection. The maximum tumor SUV, as well as the tumor to background ratios, are seen to increase with increasing time from the injection.

The subjective imaging analysis using the Schifter scale (Table 1) also showed statistically significant higher conspicuity of the tumors at later time points. This is demonstrated in Figures 3, 4. The mean rating at 1 h was 4.1 ± 1.5, at 5 h was 4.8 ± 0.7, and at 8 h was 5.0 ± 0.4. The 5 h imaging was considered superior compared to 1 h (*p* > 0.0001), 8 h was considered superior to 1 h (*p* > 0.0001), and 8 h was superior to 5 h (*p* = 0.023). The interrater correlation coefficient was substantial at 0.83.

## Discussion

The ability to assess treatment response is crucial in routine oncologic care as well as in evaluating the efficacy of new therapies. Accurate assessment of treatment response vs. failure allows for timely identification of patients who require salvage therapy. Regrettably, standard of care imaging protocols are often unable to assess glioblastoma treatment response accurately. The initial ground-breaking imaging evaluation guideline for the brain—the Macdonald criteria—were established in 1990 and was based solely on the measurement of contrast-enhancement as a surrogate for tumor size (17). Contrast-enhancement is however, non-specific, and mostly reflects the degree of extravasation of a contrast agent across a leaky blood-brain. Contrast-enhancement variation may be related to true progression, technical factors, treatment (pseudoprogression), corticosteroids, and non-malignant processes such as ischemia, seizure, infection (18).

The use of multimodal therapy with radiation and temozolomide as well as new systemic therapies involving antiangiogenic therapies (for example, bevacizumab) led to the identification of new radiological phenomena including pseudoprogression and pseudoresponse. The RANO criteria define pseudoprogression as new or increasing contrast enhancement that eventually subsides without any change in therapy (19). Pseudoresponse is a phenomenon initially identified during the trials featuring antiangiogenic therapies, like bevacizumab, which is designed to block the VEGF effect that is overexpressed in high-grade glioma tumors. The mechanism of action is related to the decreased blood supply to the tumor and improvement in the increased permeability seen in tumor vascularity (20). If only assessing the contrast-enhancing volume of the tumor, these agents are associated with high radiologic rates of response. However, the infiltrative non-enhancing tumor component is unaffected (or less affected) and eventually increases on further follow-up imaging (pseudoresponse). This scenario helps to explain the typically reduced survival benefit in trials with antiangiogenic therapy (21).

Newer response criteria (for example, the Immunotherapy Response Assessment for Neuro-Oncology) make allowances for immunotherapies (for example, nivolumab) along with live (for example, Delta-24-RDG) and attenuated viral therapies (22). Continuing to optimize imaging protocols will aid in the ability to provide accurate disease assessments and will hopefully lead to better validation of new therapies.

We prospectively assessed whether delayed (5 and 8 h post-injection) 18F-FDG imaging of untreated glioblastomas conferred a diagnostic advantage compared to the conventional imaging time point of approximately 1 h following tracer injection. Our data revealed that glioblastoma glucose metabolism, as measured by SUVmax, as well as conspicuity, as measured by L/B ratios, was significantly higher at 5 and 8 h post-injection vs. the 1 h time point. The improved conspicuity demonstrated with delayed time point imaging has the potential to be diagnostically advantageous in the post-treatment setting when evaluating for true progression of glioblastoma vs. treatment-related change can be challenging by conventional means. This data suggests a delayed imaging time point may be superior in assessing post-treated glioblastoma for true progression over pseudoprogression. These results are promising, and further investigation is warranted.

**Figure 5:**
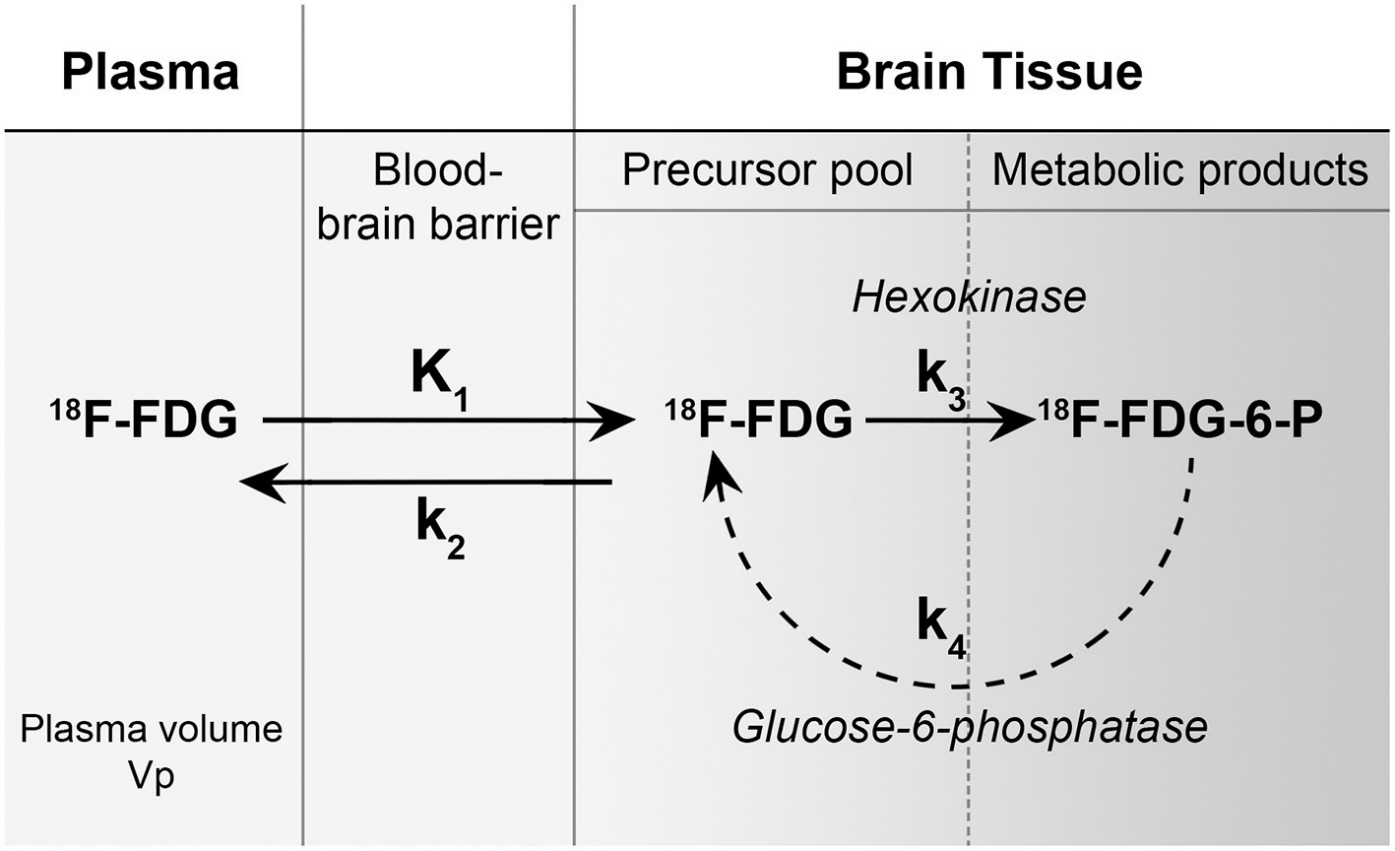
A metabolism model of 18F-FDG showing three-compartments (plasma, precursor pool in brain tissue, and metabolic product pool in brain tissue) and the corresponding forward transfer coefficients (k_1_, k_2_, k_3_, and k_4_). The forward transfer coefficient from the plasma volume into both normal brain tissue and glioblastoma (k_1_) is significantly elevated compared to other tissue (for example, skeletal muscle). It is assumed that the phosphorylation of 18F-FDG (k_3_) is a one-way process without dephosphorylation (k_4_) and therefore, also assumed that the forward transfer coefficient from the precursor pool in brain tissue back into the plasma is negligible as phosphorylated glucose and FDG are unable to cross the blood-brain barrier. [Figure adapted from reference (9)].

The rationale for the improved conspicuity of high-grade malignancies with FDG PET at later imaging time points can be explained using a three-compartment model (plasma, precursor pool in brain tissue, and metabolic product pool in brain tissue) and the corresponding forward transfer coefficients (k_1_, k_2_, k_3_, and k_4_) (Figure 5). The forward transfer coefficient from the plasma volume into both normal brain tissue and glioblastoma are significantly elevated compared to other tissue (for example, skeletal muscle). It is assumed that the phosphorylation of 18F-FDG is a one-way process without dephosphorylation and, therefore, also assumed that the forward transfer coefficient from the precursor pool in brain tissue back into the plasma is negligible as phosphorylated glucose and FDG are unable to cross the blood-brain barrier. These assumptions are valid for conventional metabolic brain imaging often performed at 30–60 min following the injection of FDG. At longer time points, however, we see differential changes with typically decreasing activity in normal brain parenchyma while glioblastoma tissue tends to increase in activity. This suggests that the k_1_ and k_3_ differences between glioblastoma and normal brain parenchyma may be negligible over a shorter examination length but significant over longer durations.

The Response Assessment in Neuro-Oncology (RANO) working group and European Association for Neuro-Oncology (EANO) collaboration toward a reformist and rational change in the imaging evaluation of gliomas by making official suggestions for PET in glioma management. These guidelines delineate the recommended application of the most studied PET agents (18F-FDG, 18F-FDOPA, 18F-FET, and 11C-MET) in glioma grading, margin assessment, treatment planning, post-therapeutic response, as well as discernment of pseudoprogression from true progression. A series of trials have also suggested that amino acid PET imaging is superior to MR imaging for each of the significant diagnostic tasks in gliomas (5). Given the relative ease of access and low cost of 18F-FDG compared to these amino acid tracers, which are not FDA approved for glioma imaging, not readily available in the US. With significant cost of over $3,000 a dose, it is important to maximize the diagnostic utility of 18F-FDG.

This study has multiple limitations. All 16 patients were isocitrate dehydrogenase (IDH) wild type, and thus it is uncertain whether these results would generalize to patients with IDH-mutant genes. However, IDH wild-type glioblastomas constitute ~ 90% of glioblastomas and thus the subset not represented in this study is a small fraction of the overall glioblastoma population (23). The study is of modest size, but to our knowledge, represents the largest study of glioblastoma patients at the same phase of treatment with fixed delayed imaging time points and provided statistically significant findings. Prior studies included smaller sizes of glioblastoma patients, sometimes included both treated and untreated patients, and did not consistently report the post-injection time for the glioblastoma patients. Patients were instructed to fast and then to follow a specific set of dietary restrictions between the imaging time points, but were not supervised in their choices. Thus, it is uncertain whether some apparent time-related effects could have been masked or attenuated by this variable. It would have been ideal to more closely supervise the patients' dietary choices before and during the examinations and also to check their blood glucose before each imaging session to understand whether these variables lead to important variations in findings. However, these elements would lead to extra cost and complexity to the research, and more tightly controlling these parameters could also potentially limit the generalizability of the results to routine clinical use where these elements could not be routinely controlled in an outpatient ambulatory population. The routine clinical application of the delayed PET imaging technique may be operationally challenging for some imaging centers and patients in poor clinical condition; however, our imaging protocol was optimized for research purposes. A diagnostic protocol relying solely on imaging at approximately 5 h after imaging is likely adequate to provide actionable complementary information to MR imaging, especially given the sensitivity of modern PET detectors. The table time of the 5-h imaging session in this study was 20 min, which provided adequate count statistics and subjective quality assessment. Future work may identify that shorter imaging times are sufficient, particularly given work on novel reconstruction algorithms for PET imaging with lower radiopharmaceutical doses (24, 25).

## Conclusion

We prospectively revealed that glioblastoma conspicuity with FDG PET, as measured by SUVmax, as well as conspicuity, as measured by L/B ratios, was significantly greater at 5 and 8 h post-injection vs. a 1-h time point. We speculate from these results that the use of delayed FDG PET may provide improvements in the accuracy of FDG PET in assessing glioblastoma treatment response (true progression vs. pseudoprogression). Future work will be necessary to validate whether the objective and subjective improvement in the contrast between neoplastic and healthy brain tissue at delayed imaging time points also provide improvement in differentiation between true progression and pseudoprogression in the setting of abnormal post-treatment enhancement. These results support continued diagnostic utilization of delayed FDG PET imaging at our center. These results should also serve to aid in the design of future studies assessing the comparative diagnostic value of amino acid PET radiopharmaceuticals given their considerably higher cost compared to FDG.

## Data Availability Statement

The raw data supporting the conclusions of this article will be made available by the authors, without undue reservation.

## Ethics Statement

The studies involving human participants were reviewed and approved by the University of Texas MD Anderson Cancer Center Institutional Review Committee. The patients/participants provided their written informed consent to participate in this study.

## Author Contributions

JJ, ER, SP, BC, and OM: experimental design. JJ, ER, BC, OM, MC, and MG-M: study implementation. JJ, OM, H-LL, MC, and MG-M: data analysis and interpretation. All authors were involved in the manuscript preparation.

## Funding

This study was funded by the Diagnostic Imaging Clinical Research Committee of the University of Texas MD Anderson Cancer Center. Funds were received for open access publication fees through my institution.

## Conflict of Interest

The authors declare that the research was conducted in the absence of any commercial or financial relationships that could be construed as a potential conflict of interest.

## Publisher's Note

All claims expressed in this article are solely those of the authors and do not necessarily represent those of their affiliated organizations, or those of the publisher, the editors and the reviewers. Any product that may be evaluated in this article, or claim that may be made by its manufacturer, is not guaranteed or endorsed by the publisher.
